# A novel platform for comprehensive CMR examination in a clinically feasible scan time

**DOI:** 10.1186/1532-429X-16-S1-W10

**Published:** 2014-01-16

**Authors:** Joëlle K Barral, William R Overall, Michelle M Nystrom, Xue Feng, R Reeve Ingle, Kenneth O Johnson, Bob S Hu, Juan M Santos

**Affiliations:** 1 HeartVista, Inc., Menlo Park, California, USA

## Background

Cardiac magnetic resonance imaging has been shown to be one of the best technologies for the evaluation of cardiovascular pathologies. However, its widespread adoption has been held back by the lack of software infrastructure and specific pulse sequences that can provide a feasibly quick, comprehensive cardiac examination. We have developed an intuitive software package that can perform a comprehensive CMR examination in 30 to 45 minutes.

## Methods

Our clinical protocol is built within the RTHawk real-time environment, which interfaces with a GE scanner and allows for intuitive localization and real-time scanner interaction. It comprises all the applications needed for a comprehensive CMR examination: real-time localization, left ventricular function, black blood, quantitative valvular flow, myocardial perfusion, delayed enhancement, and coronary angiography. Free-breathing, not ECG-gated options exist for the applications that assess ventricular function and flow. These work well even with uncooperative patients and are robust against arrhythmia.

## Results

**Figure 1:**
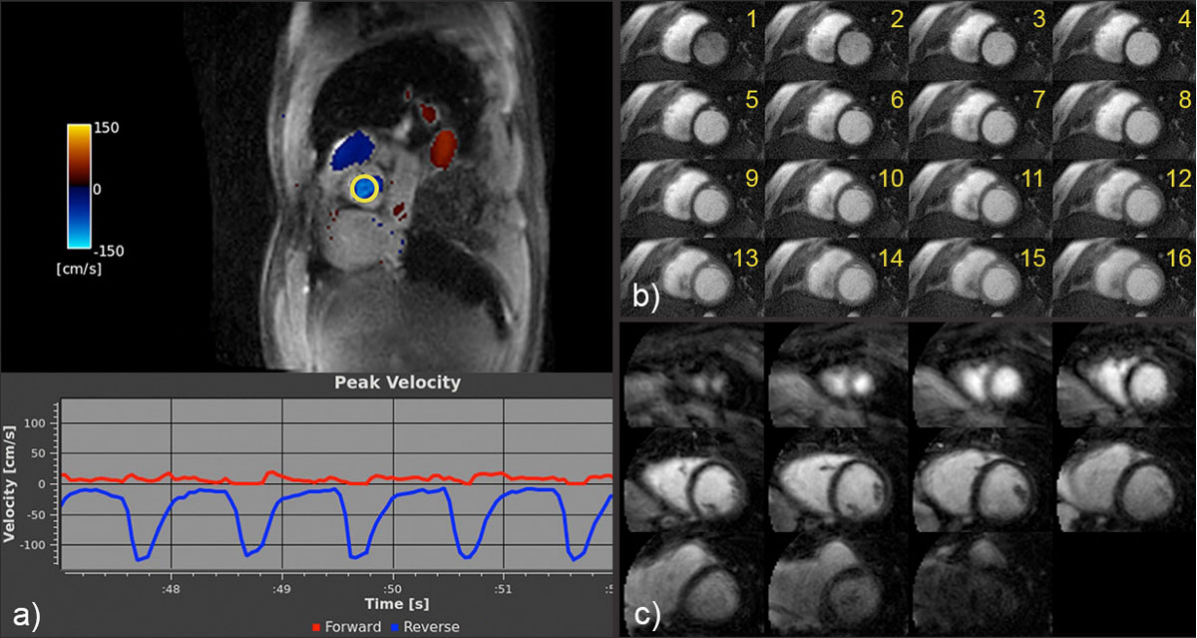
**Examples of patient images obtained using our cardiac protocol: (a) real-time color flow (map and plot in the aortic valve ROI), (b) 2D perfusion, (c) 3D delayed-enhancement**.

**Figure 2:**
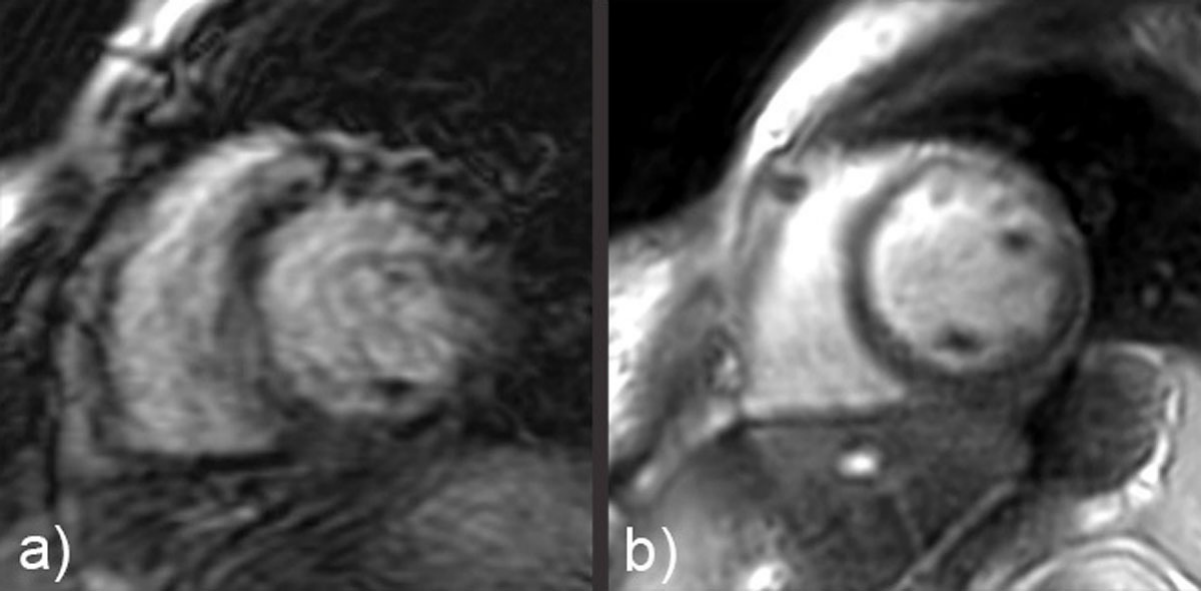
**Comparison of images obtained during arrhythmia using (a) a traditional breath-held SSFP CINE application and (b) our non-gated, free-breathing SSFP application**.

Figure 1 provides example images acquired using our protocol in patients under local IRB approval and consent. Figure 1a demonstrates a color flow short-axis view and the peak velocity plot of the selected region of interest (ROI) in the aortic valve, both of which were acquired and displayed in real time (flow encoded through-plane, 22 fps effective temporal resolution). The intuitive scan interaction allows for easy localization of the valve. Figure 1b displays the first 16 temporal frames of one slice of a 3-slice perfusion acquisition (1.5 mm in-plane resolution, 1 temporal frame per heartbeat, free breathing). Real-time imaging is used to track the contrast bolus in an area of interest prior to starting the scan. Figure 1c shows all 11 slices of a 3D delayed-enhancement scan (1.8 × 1.8 × 8 mm3 resolution, 8-heartbeat breath-hold). Non-Cartesian SPIRiT reconstruction is implemented for both the perfusion and delayed-enhancement applications, with a reconstruction time of a few seconds on the RTHawk scan console (dual quad-core Xeon, 64GB). Figure 2 compares similar short-axis views obtained on a patient suffering from arrhythmia, either (a) using a traditional CINE SSFP sequence (14 slices, 20 cardiac phases, 1.3 × 2.6 mm2 in-plane resolution, 17-heartbeat breath-hold per slice) or (b) using our non-gated multi-slice SSFP sequence (12 slices, 50 cardiac phases, 2.3 × 2.3 mm2 resolution, 24-heartbeat scan duration, free breathing). Our non-gated, free-breathing sequence is immune to arrhythmia, whereas the traditional approach fails.

## Conclusions

We have implemented a comprehensive cardiac MR system, which performs an examination in less than 45 minutes and provides alternative solutions to traditional sequences when a patient suffers from arrhythmia or is unable to hold his breath. Initial clinical results are encouraging.

